# Supplementary material for: The Transposition of Insertion Sequences in Sigma-Factor- and LysR-Deficient Mutants of Deinococcus geothermalis
Source: Microorganisms. 2024 Feb 4;12(2):328. doi: 10.3390/microorganisms12020328 (PMC10892881; doi:10.3390/microorganisms12020328)
Supplement: Supplementary file 1 [file microorganisms-12-00328-s001.zip › microorganisms-2853242-supplementary.pdf]

Supplementary data

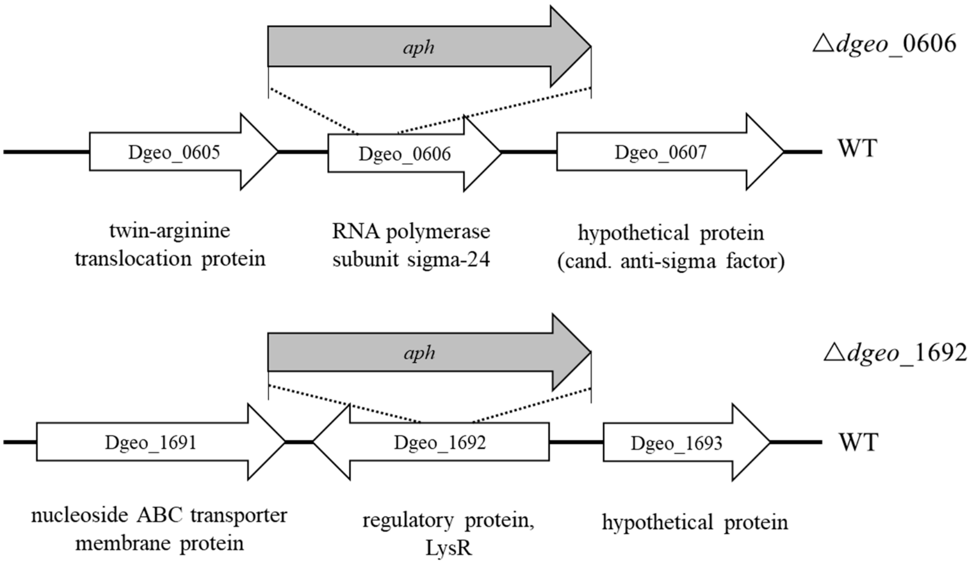

Figure S1. Scheme for construction of the Δ*dgeo*_0606 and Δ*dgeo*_1692 mutants by homologous recombination using kanamycin-resistant selection using pKATAph vector which was explained in materials and methods. *aph* is a kanamycin-resistant gene.

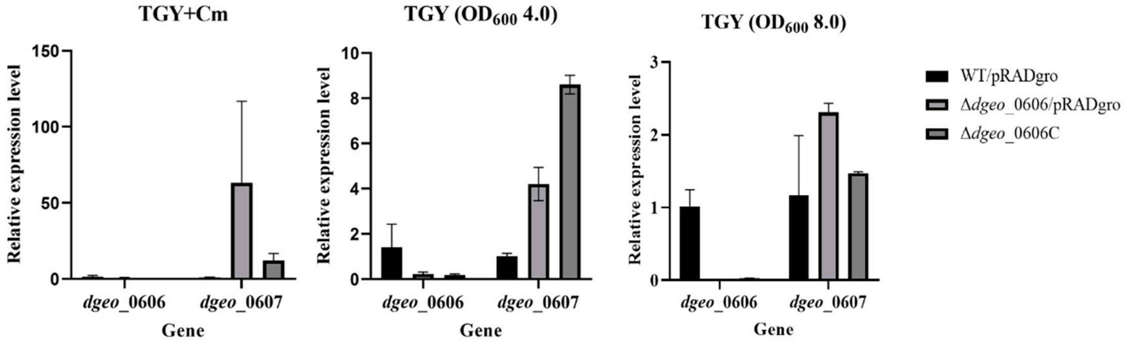

Figure S2. Expression levels of *dgeo*_0606 and *dgeo*_0607 by qRT-PCR at different growth condition (chloramphenicol present or absent) and phases (OD_{600} of 4.0 or 8.0). Chloramphenicol was added with 3 μg/mL. The relative expression levels were normalized to 1.0 of *dgeo*_0606 expression level in WT/pRADgro strain.

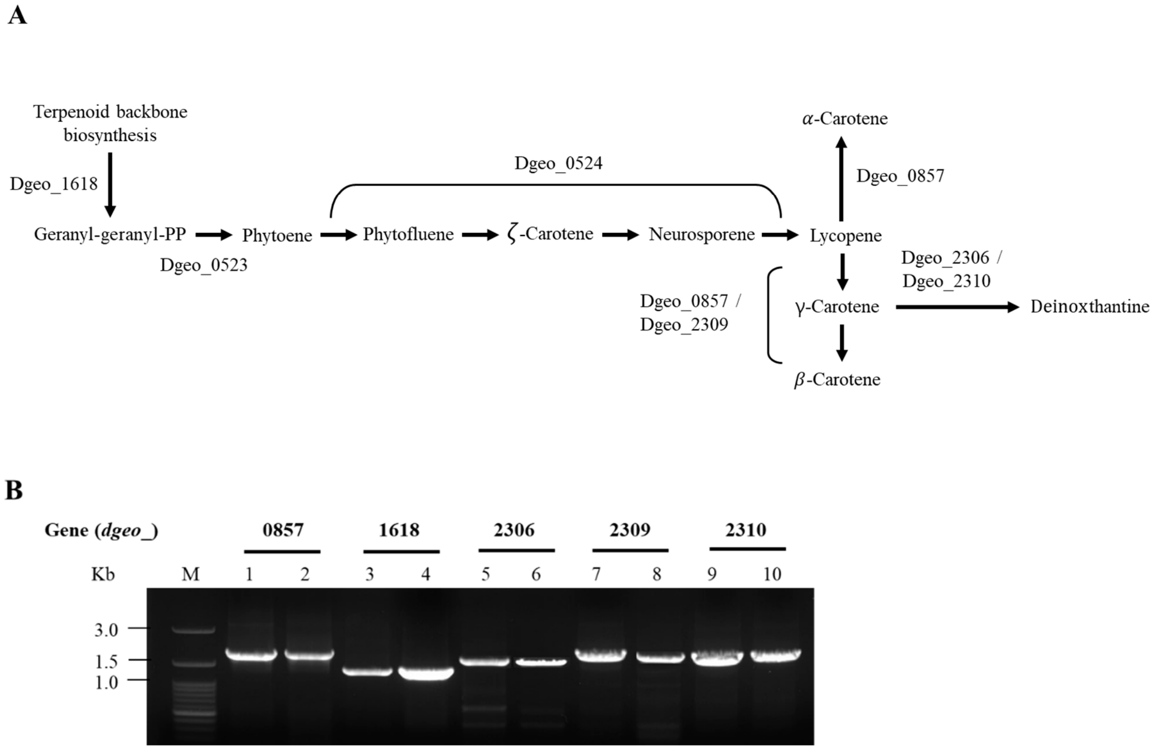

Figure S3. (**A**) Scheme of carotenoid biosynthesis pathway in *D. geothermalis*. (**B**) PCR detection of five carotenoid pathway involved genes (except *dgeo*_0523 and *dgeo*_0524) for IS transposition in Δ*dgeo*_1692*hw*.

Table S1. Analysis of specific IS type inserted in transposition loci of IS in Δ*dgeo*_0606*gw* and Δ*dgeo*_1692*gw* mutants under gamma irradiation.

Strain	Non-Pigments	OD_{600}	Gamma -Radiation	Transposase Type	Direction	Loci
△*dgeo*_0606	*gw*1/6/8	4.0	5 kGy	IS*5* family IS*Dge6*	–	8th of *dgeo*_0524
△*dgeo*_0606	*gw*4	4.0	5 kGy	IS*5* family IS*Dge6*	+	319th of *dgeo*_0524
△*dgeo*_0606	*gw*3/9	2.0	5 kGy	IS*1* family IS*Dge2*	–	1289th of *dgeo*_0524
△*dgeo*_1692	*gw*1	4.0	5 kGy	IS*5* family IS*Dge7*	–	1673rd of *dgeo*_0524
△*dgeo*_1692	*gw*2	4.0	5 kGy	IS*701* family IS*Dge5*	+	612nd of *dgeo*_0524

Table S2. Analysis of specific IS type inserted in transposition loci of IS in WT*hw* and Δ*dgeo*_0606*hw* mutant under hydrogen peroxide treatment.

**Strain**	**Non-Pigments**	$\mathbf{OD_{600}}$	$\mathbf{H_2O_2}$ **(mM)**	**Transposase Type**	**Direction**	**Loci**
WT	*hw*1/2	2.0	80	IS*5* family IS*Dge6*	−	8th of *dgeo*_0524
△*dgeo*_0606	*hw*1	2.0	80	IS*5* family IS*Dge6*	+	723rd of *dgeo*_0524
△*dgeo*_0606	*hw*2	2.0	100	IS*5* family IS*Dge7*	−	1403rd of *dgeo*_0524
△*dgeo*_0606	*hw*3	2.0	100	IS*701* family IS*Dge5*	−	1450th of *dgeo*_0524
